# Patient Safety Culture: A Healthcare Provider’s Prospect

**DOI:** 10.7759/cureus.49989

**Published:** 2023-12-05

**Authors:** Sajid Hameed, Ayesha Humayun, Muhammad Yaqoob, Muhammad H Rehm

**Affiliations:** 1 Public Health, University Institute of Public Health, Faculty of Allied Health Sciences, The University of Lahore, Lahore, PAK; 2 Public Health and Community Medicine, Shaikh Zayed Postgraduate Medical Institute, Lahore, PAK; 3 Community Medicine, Sahiwal Medical College, Sahiwal, PAK

**Keywords:** pakistan, lahore, tertiary care hospitals, safety, patients

## Abstract

Introduction and objective: Safe care is a challenge around the globe, especially in developing countries. In resource-limited settings achieving patient safety is an additional complexity. Patient safety is now considered a significant public health concern worldwide. Despite a vital role in delivering quality care, little attention has been given to describe healthcare professionals' perceptions of the patient safety culture in Pakistan. This study aimed to assess the patient safety culture at a tertiary care public hospital in Lahore from the perspectives of doctors and nurses.

Methods: During this cross-sectional study, data were collected from 290 nurses and doctors using a validated safety assessment survey tool of the Hospital Survey of Patient Safety Culture (HSOPSC). The respondent's demographic characteristics and study variables influencing patient safety culture were presented, and a chi-square test was applied to identify the variables influencing patient safety.

Results: A total of 114 medical doctors (39.3%) and 176 registered nurses (60.7%) participated in assessing patient safety culture (PSC) across tertiary care public healthcare centers in Lahore. The dimensions of organizational learning and continuous improvement (90.6%) and teamwork within units (86.6%) were the highest. Other dimensions include feedback and communication about the error (71.8%), teamwork across units (74.9%), management support for patient safety (67.3%), supervisor/manager expectations, and actions promoting patient safety (64.6%), communication openness (64.5%), overall perceptions of patient safety (65.3%), frequency of events reported (58.7%), and handoffs and transitions (60.9%) showed moderate status. The dimensions of staffing (35.8%) and non-punitive response to errors (39.1%) had the lowest score.

Conclusions: The present public hospital survey results revealed that medical staff working in a healthcare setting have a less positive perception of patient safety culture.

## Introduction

Patient safety is a serious public health issue affecting countries around the globe [1]. The accurate estimation of patient safety issues and adverse events in hospitals is scarce, particularly in developing and transitional countries where patients suffer injuries and disabilities or death due to malpractice and unsafe patient care [2,3]. The delivery of safe medical care to patients is considered one of the essential tenets in establishing a patient safety culture and is a critical element of healthcare quality [4]. The patient safety culture of a healthcare organization is a cumulative product of medical professionals, healthcare managers, individuals’ attitudes and perceptions, competencies, and behaviors that determine the commitment and proficiency of an organization and management. Due to the potential impact of common deficiencies, including poor communication, leadership, weak teamwork, lack of error reporting, and insufficient staff knowledge of patient safety structure, the organization's existing safety culture is crucial. It, therefore, needs urgent attention [5]. Patient safety culture assessment surveys allow medical professionals and healthcare management to identify weaknesses and strengths of the existing safety culture. It can be determined by multiple factors involved in the healthcare system of any health organization and can support the reduction of the occurrence of adverse events in patients [6]. Remarkably, international accreditation organizations require patient safety assessment surveys as a fundamental component to better understand overall organizational perception related to patient safety [7]. Keeping in account, several hospitals around the globe are assessing patient safety practices using different tools to promote patient care. In this subject, the World Health Organization (WHO) and previous studies have recommended the Hospital Survey on Patient Safety Culture (HSOPSC) as a reliable and most frequent tool to assess patient safety culture and to understand different aspects of patient care [8,9].

Owing importance of patient safety culture, a lot of studies have been conducted in the United States and European countries; however, information related to patient care is scarce in Pakistan [10-13]. Being a developing country, Pakistan may encounter several adverse events that may be reported or not reported [14]. The reasons might be due to the developing nature of Pakistan's healthcare system and organizations. As in many countries, in Pakistan, the number of malpractice litigations against healthcare providers has increased dramatically [15]. Hence, the necessity of initiating patient safety culture in Pakistani healthcare systems should be raised. Despite a vital role in delivering quality care, little attention has been given to describing healthcare professionals' perceptions and expectations of the patient safety culture. Therefore, it is imperative to explore the beliefs and attitudes of healthcare staff and management to raise their awareness and strengthen patient care interventions. Furthermore, initiating and maintaining vital patient safety care in a healthcare organization is directly linked to better-performing healthcare organizations and subsequently establishing a better healthcare system. A piece of up-to-date information on patient safety within healthcare organizations may be provided to healthcare leaders and policymakers to develop and improve quality care interventions [16]. Administrators, managers, and policymakers alike will reap the benefits of improving patient safety culture in enhanced quality, improved patient outcomes, reduced errors, and a more cost-effective healthcare system. It is, therefore, imperative to explore the knowledge and perception of doctors and nurses about their patient safety practices to ensure success in providing patients with the safest and highest quality of care. The present study aimed to assess the patient safety culture at a tertiary care public hospital in Lahore from the perspectives of doctors and nurses using the HSOPSC.

## Materials and methods

Study design and setting

The cross-sectional study was carried out to assess the patient safety culture at a public tertiary care hospital (Services Hospital Lahore) from November 2019 to November 2020. The tertiary care hospital was included because it caters to a large number of patients, possesses complex processes, consists of multiple departments, has an extensive infrastructure, and encompasses huge human resources. All these factors make the tertiary care hospital a compound facility and increase the likelihood of human errors and adverse events. The study included a tertiary care public hospital with a functional experience of 15 years, a high patient turnover with more than 300 bed-capacity, and an accreditation with the Punjab Healthcare Commission (PHCC). Primary and secondary hospitals non-accredited with PHCC were excluded. This cross-sectional study collected data from 290 nurses and doctors of one public hospital. These participants were selected through a systematic random sampling method according to inclusion criteria. As per the sampling technique, 30% of healthcare professionals, including nurses and doctors, were interviewed using the HSOPSC tool. Before starting the survey, healthcare practitioners were asked for informed consent.

Inclusion and exclusion criteria

Doctors and nurses working at the specified tertiary care public hospital in Lahore and participants who have agreed to provide informed consent to participate were included in the study. In contrast, non-medical staff members (e.g., maintenance staff and support staff) who are not directly involved in patient care, and individuals who do not provide informed consent to participate were excluded from this study.

Survey tool/research instrument

A validated safety assessment survey tool known as the Hospital Survey of Patient Safety Culture (HSOPSC) developed by the Agency for Healthcare Research and Quality (AHRQ) was used to get information from nurses and doctors and subsequently assess patient safety culture. These safety culture dimensions were categorized into different units at the hospital level and outcome measures. This tool was comprised of all dimensions of patient safety culture, including communication openness, feedback and communication about errors, staffing, frequency of event reporting, supervisor expectation and action promoting safety, handoffs and transitions, management support for patient safety, non-punitive response to error, organizational learning and continuous improvement, teamwork, and overall perception of patient safety. All instructions and queries were written in English, and targeted participants could speak and write in English.

Data collection/questionnaire

After receiving proper informed consent, the adopted survey questionnaire was distributed to nurses and doctors through personal delivery in a tertiary care hospital. All dimensions of patient safety consisted of three or four questions. These questions were related to demographic characteristics, preliminary work and responsibilities of targeted participants, regular practices of the hospital, staff satisfaction, management cooperation, hospital working environment, monitoring and reporting of an adverse event, patient safety grade, and promotion of patient safety. The proforma was self-administered. All these questions were assessed on a five-point Likert scale with scores ranging from 1 to 5 as 1 (strongly disagree), 2 (disagree), 3 (neither/neutral), 4 (agree), and 5 (strongly agree).

Data analysis

Data were entered and analyzed for each dimension by calculating a score that represents the average percentage of positive and negative responses. The questions with a positive formulation and answers like "agreed" and "strongly agreed" were considered positive. In contrast, answers like "disagree" and "strongly disagree" were deemed negative for patient safety culture. The dimension with a score of ≤50% was considered to be improved, while the dimension with a score of ≥75% was considered developed [17]. Descriptive statistics were done to explore the association of demographic characteristics and other study variables influencing patient safety culture. The respondent's demographic characteristics were presented using descriptive statistics. Based on user guide instructions published by AHRQ, frequencies and positive response rates were determined [17]. A chi-square test was applied to identify the variable influence on patient safety care (SPSS software version 21; Armonk, NY: IBM Corp.) at the p ≤ 0.05 significant level.

## Results

Socio-demographic characteristics of respondents

In the present study, 114 medical doctors (39.3%) and 176 registered nurses (60.7%) participated to assess patient safety culture (PSC) across a tertiary care public hospital in Lahore. Most of the respondents worked in medicine unit (n = 155 {53.4%}) followed by surgery unit (n = 28 {9.6%}), emergency unit (n = 19 {6.5%}), ICU (n = 16 {5.5%}), pediatrics unit (n = 15 {5.1%}), obstetrics unit (n = 10 {3.4%}), dermatology unit (n = 3 {1.03%}), psychiatric, radiology, nursing management, cardiology and orthopedics units (each n = 2 {0.68%}), and anesthesiology and ophthalmology units (each n = 1 {0.34%}), while 11.1% (n = 32) of participants did not response. Among these, most of the respondents (n = 147 {50.6%}) had less than one year of professional experience followed by one to five years (n = 81 {27.9%}), 6-10 years (n = 24 {8.3%}), 11-15 years (n = 21 {7.2%}), and 16-20 years (n = 10 {3.4%}), while few respondents (n = 7 {2.4%}) had 21 years or more professional experience.

Among all, most of respondents were working from few months (n = 156 {53.7%}) followed by one to five years (n = 75 {25.8%}), 6-10 years (n = 21 {7.2%}), 11-15 years (n = 21 {7.2%}), and 16-20 years (n = 10 {3.4%}) with least numbers of respondents in 21 years or above category (n = 7 {2.4%}). Most of the respondents (n = 102 {35.2%}) worked 40-59 hours per week followed by 60-79 hours per week (n = 76 {26.2%}), 20-39 hours per week (n = 52 {17.9%}), 80-99 hours per week (n = 27 {9.3%}), and 100 hours per week (n = 20 {6.9%}) with minimum working hours of less than 20 hours per week (n = 22 {5.4%}). Most of the doctors and registered nurses had direct interaction with patients (n = 269 {92.7%}) compared to those who had indirect contact with patients (n = 21 {7.8%}). Considering the specialty of all respondents, 47.9% of respondents (n = 139) were working for less than one year, 30.7% of respondents (n = 89) were working from one to five years, 9.6% of respondents (n = 28) were working from less than six to 10 years, 5.5% of respondents (n = 16) were working from 11 to 15 years, 3.7% of respondents (n = 11) were working from 21 or above years, and 2.4% of respondents (n = 7) were working from 16 to 20 years (Table 1). Among these socio-demographic determinates, work in various units and professional experience have a significant association (p ≤ 0.05) with PSC.

**Table 1:** Demographic characteristics of the respondents from public tertiary care hospitals in Lahore. *P-value was considered not significant. **P-value ≤ 0.001 was considered very highly significant. ***P-value ≤ 0.01 was considered highly significant. P-value ≤ 0.05 was considered significant. N: number of respondents; 95% CI: confidence interval at 0.05 significance level

Characteristics	Frequency (n)	Percentage (%)	p-Value (χ2)
Designation (mean±SEM: 1.6±0.02, 95% CI: 1.55-1.66)			
Medical doctor	114	39.3	0.499^*^
Registered nurse	176	60.7	
What is your primary work area or unit in this hospital (mean±SEM: 3.4±0.19, 95% CI: 3.04-3.82)			
Medicine	155	53.4	0.000**
Surgery	28	9.6	
Obstetrics	10	3.4	
Pediatrics	15	5.1	
Emergency	19	6.5	
ICU	16	5.5	
Psychiatry/mental health	2	0.68	
Radiology	2	0.68	
Anesthesiology	1	0.34	
Nursing management	2	0.68	
Ophthalmology	1	0.34	
Dermatology	3	1.03	
Cardiology	2	0.68	
Orthopedics	2	0.68	
No specific	32	11.1	
How long have you worked in this hospital? (mean±SEM: 1.9±0.07, 95% CI: 1.80-2.19)			
Less than 1 year	147	50.6	0.000**
1-5 years	81	27.9	
6-10 years	24	8.3	
11-15 years	14	4.8	
16-20 years	14	4.8	
21 years or more	10	3.4	
How long have you worked in your current hospital work area/unit? (mean±SEM: 1.8±0.07, 95% CI: 1.72-2.02)			
Less than 1 year	156	53.7	0.000**
1-5 years	75	25.8	
6-10 years	21	7.2	
11-15 years	21	7.2	
16-20 years	10	3.4	
21 years or more	7	2.4	
Typically, how many hours per week do you work in this hospital? (mean±SEM: 3.3±0.07, 95% CI: 3.24-3.52)			
Less than 20 hours per week	13	4.4	0.319^*^
20-39 hours per week	52	17.9	
40-59 hours per week	102	35.2	
60-79 hours per week	76	26.2	
80-99 hours per week	27	9.3	
100 hours per week or more	20	6.9	
In your staff position, do you typically have direct interaction or contact with patients? (mean±SEM: 1.0±0.01, 95% CI: 1.04-1.10)			
Direct	269	92.7	0.063^*^
Indirect	21	7.2	
How long have you worked in your current specialty or profession? (mean±SEM: 1.9±0.07, 95% CI: 1.80-2.09)			
Less than 1 year	139	47.9	0.001***
1-5 years	89	30.7	
6-10 years	28	9.6	
11-15 years	16	5.5	
16-20 years	7	2.4	
21 years or more	11	3.7	

Composite items of patient safety culture

Among participants, 74.8% of respondents reported a positive support rate at the workplace. Out of 290 participants, 53.7% of respondents indicated staff availability to lead with workload within the healthcare unit. Most of the respondents (80.6%) worked to complete different tasks. Only 79.6% of respondents indicated that people treated each other with respect within the unit. A total of 193 respondents (66.5%) reported that all staff worked longer to improve patient safety practices with active engagements (82.7%). Only 41.1% of respondents indicated that staff was better for patient care. Few respondents (38.9%) considered that their mistakes could hold against them. However, some respondents (55.5%) believed that their mistakes have led to positive changes. A total of 152 respondents (52.4%) indicated that the occurrence of any serious adverse event might depend upon luck. Most of the staff (60%) offered help during burden within the unit. About 79.3% of respondents indicated that improvement in patient safety was evaluated by its effective change. Similarly, a total of 226 respondents (77.9%) reported that patient safety was their preference and never sacrificed to get completion of work quickly (Table 2).

**Table 2:** Frequency percentage of positive, neutral, and negative responses of respondents from public tertiary care hospitals in Lahore. *P-value was considered not significant. **P-value ≤ 0.01 was considered highly significant. ***P-value ≤ 0.001 was considered very highly significant. P-value ≤ 0.05 was considered significant. N: number of respondents; 95% CI: confidence interval at 0.05 significance level

Items	Mean±SEM	95% CI (mean)	Positive, n (%)	Neutral, n (%)	Negative, n (%)	p-Value (χ2)
People support one another in this unit	3.9±0.05	3.82-4.03	224 (77.2)	42 (14.5)	24 (8.2)	0.326*
We have enough staff to handle the workload	3.2±0.06	3.14-3.41	156 (53.7)	39 (13.4)	95 (32.7)	0.604*
When a lot of work needs to be done quickly, we work together as a team to get the work done	3.9±0.04	3.86-4.04	234 (80.6)	34 (11.7)	19 (6.5)	0.136*
In this unit, people treat each other with respect	4.0±0.05	3.91-4.11	231 (79.6)	43 (14.8)	16 (5.5)	0.184*
Staff in this unit work longer hours than is best for patient care	3.6±0.06	3.54-3.78	193 (66.5)	47 (16.2)	50 (17.2)	0.162*
We are actively doing things to improve patient safety	4.0±0.04	3.96-4.13	240 (82.7)	44 (15.2)	6 (2.1)	0.642*
We use more agency/temporary staff than is best for patient care	3.0±0.06	2.95-3.20	119 (41.1)	69 (23.7)	102 (35.2)	0.179*
Staff feel like their mistakes are held against them	3.0±0.05	2.98-3.20	113 (38.9)	87 (30)	90 (31)	0.835*
Mistakes have led to positive changes here	3.4±0.04	3.34-3.53	161 (55.5)	87 (30)	42 (14.4)	0.292*
It is just by chance that more serious mistakes don’t happen around here	3.3±0.05	3.20-3.44	152 (52.4)	65 (22.4)	73 (25.2)	0.073*
When one area in this unit gets really busy, others help out	3.4±0.06	3.28-3.55	174 (60)	46 (15.8)	70 (24.1)	0.127*
When an event is reported, it feels like the person is being written up, not the problem	3.2±0.06	3.11-3.36	144 (49.6)	66 (22.7)	80 (27.5)	0.101*
After we make changes to improve patient safety, we evaluate their effectiveness	3.8±0.04	3.77-3.93	230 (79.3)	48 (16.5)	12 (4.1)	0.003**
We work in "crisis mode" trying to do too much, too quickly	3.6±0.05	3.50-3.73	198 (68.3)	46 (15.8)	46 (15.8)	0.071*
Patient safety is never sacrificed to get more work done	3.9±0.05	3.80-4.01	226 (77.9)	38 (13.1)	26 (8.9)	0.173*
Staff worry that mistakes they make are kept in their personnel file	3.2±0.05	3.14-3.37	132 (45.5)	80 (27.5)	78 (26.8)	0.000***
We have patient safety problems in this unit	2.6±0.06	2.56-2.81	83 (28.6)	58 (20)	149 (51.4)	0.080*
Our procedures and systems are good at preventing errors from happening	3.4±0.05	3.35-3.59	174 (60)	58 (20)	58 (20)	0.000***
My supervisor/manager says a good word when he/she sees a job done according to established patient safety procedures	3.9±0.05	3.80-3.99	227 (78.2)	40 (13.7)	23 (7.9)	0.148*
My supervisor/manager seriously considers staff suggestions for improving patient safety	3.7±0.05	3.64-3.84	211 (72.7)	49 (16.8)	30 (10.3)	0.317*
Whenever pressure builds up, my supervisor/manager wants us to work faster, even if it means taking shortcuts	3.1±0.05	3.04-3.28	136 (46.8)	62 (21.4)	92 (31.7)	0.101*
My supervisor/manager overlooks patient safety problems that happen over and over	3.1±0.06	3.07-3.28	143 (49.3)	53 (18.3)	94 (32.4)	0.416*
We are given feedback about changes put into place based on event reports	3.2±0.06	3.12-3.39	123 (42.4)	103 (35.5)	64 (22.1)	0.000***
Staff will freely speak up if they see something that may negatively affect patient care	3.5±0.07	3.41-3.71	163 (56.2)	59 (20.3)	68 (23.4)	0.006**
We are informed about errors that happen in this unit	3.7±0.06	3.61-3.88	176 (60.6)	73 (25.2)	41 (14.1)	0.419*
Staff feels free to question the decisions or actions of those with more authority	3.1±0.07	3.02-3.32	131 (45.2)	59 (20.3)	100 (34.5)	0.064*
In this unit, we discuss ways to prevent errors from happening again	3.7±0.06	3.61-3.86	152 (52.4)	69 (23.7)	42 (14.4)	0.219*
Staff are afraid to ask questions when something does not seem right	2.5±0.07	2.41-2.70	72 (24.8)	73 (25.2)	145 (50)	0.097*
When a mistake is made, but is caught and corrected before affecting the patient, how often is this reported?	3.3±0.06	3.17-3.34	128 (44.1)	93 (32.1)	69 (23.7)	0.673*
When a mistake is made, but has no potential to harm the patient, how often is this reported?	3.1±0.06	3.00-3.26	108 (37.2)	90 (31.1)	92 (31.7)	0.031*
When a mistake is made that could harm the patient, but does not, how often is this reported?	3.2±0.07	3.13-3.41	123 (42.4)	75 (25.8)	92 (31.7)	0.111*
Please give your work area/unit in this hospital an overall grade on patient safety	2.1±0.05	2.05-2.25	9 (3.1)	95 (32.7)	186 (64.1)	0.000***
Hospital management provides a work climate that promotes patient safety	3.6±0.05	3.53-3.75	202 (69.6)	40 (13.7)	48 (16.5)	0.076*
Hospital units do not coordinate well with each other	2.7±0.06	2.58-2.83	96 (33.1)	32 (11.1)	162 (55.8)	0.131*
Things “fall between the cracks” when transferring patients from one unit to another	3.0±0.06	2.94-3.18	125 (43.1)	72 (24.8)	98 (33.7)	0.195*
There is good cooperation among hospital units that need to work together	3.5±0.05	3.46-3.69	201 (69.3)	46 (15.8)	43 (14.8)	0.019*
Important patient care information is often lost during shift changes	2.5±0.06	2.46-2.71	70 (24.1)	71 (24.4)	149 (51.4)	0.016*
It is often unpleasant to work with staff from other hospital units	2.7±0.05	2.64-2.87	73 (25.2)	82 (28.3)	135 (46.5)	0.227*
Problems often occur in the exchange of information across hospital units	3.0±0.05	2.88-3.11	109 (37.5)	85 (29.3)	96 (33.1)	0.410*
The actions of hospital management show that patient safety is a top priority	3.6±0.06	3.50-3.75	205 (70.6)	30 (10.3)	55 (18.9)	0.009**
Hospital management seems interested in patient safety only after an adverse event happens	3.2±0.06	3.11-3.38	148 (51.1)	34 (11.7)	108 (37.2)	0.658*
Hospital units work well together to provide the best care for patients	3.8±0.04	3.70-3.89	220 (75.8)	45 (15.5)	25 (8.6)	0.002**
Shift changes are problematic for patients in this hospital	3.0±0.06	2.92-3.18	128 (44.1)	51 (17.5)	201 (69.3)	0.379*

Some staff (45.5%) were worried that mistakes would be kept in their personnel file. Few respondents (28.6%) showed severe concern about patient safety within the unit. Most of the respondents (60%) were satisfied that the hospital has sound systems and procedures to prevent an error or adverse event. Most of the respondents (78.2%) indicated that their manager or supervisor always appreciated our contribution to improving patient safety culture. Few respondents (72.7%) indicated that their supervisors or managers imposed on working faster even utilizing shortcuts. A total of 143 respondents (49.3%) reported that their supervisors or managers overlooked patient safety problems within the unit. Few respondents (42.4%) indicated that they always provided feedback about changes after any adverse event. A total of 163 (56.2%) respondents stated that they were free to speak on the happening of any adverse event and its negative effects on patient safety. Most of the respondents (60.6%) were aware of any damaging error within the unit (Table 2).

Only 45.2% of respondents were free to ask about decisions and actions from authorities within the unit. Among all, 52.4% of respondents reported that they were discussed and offered the practices to overcome adverse events. Few respondents (24.8%) showed fear of asking any question related to any adverse event. The positive response to correcting an error that affects patient safety was low (<45%). Notably, a deficient number of respondents gave an overall positive grade on the unit's patient safety practice (3.1%). Most respondents (69.6%) were satisfied that hospital management provided a comfortable work climate to promote patient safety. Few respondents (33.1%) indicated that hospital units do not have supportive coordination. Few respondents (43.1%) raised concerns that important information related to patient care was often lost during shift change. Most of the respondents (69.3%) showed good cooperation among all units of hospitals. Few respondents (24.1%) indicated that important information related to patient care might be lost during shift changes within the unit. Also, the rate of unwillingness to work with staff from other units was very low (25.2%). Most of the respondents (70.6%) indicated that hospital management has patient safety as a top priority, with an interest of 51.1% after an adverse event (Table 2).

**Figure 1:**
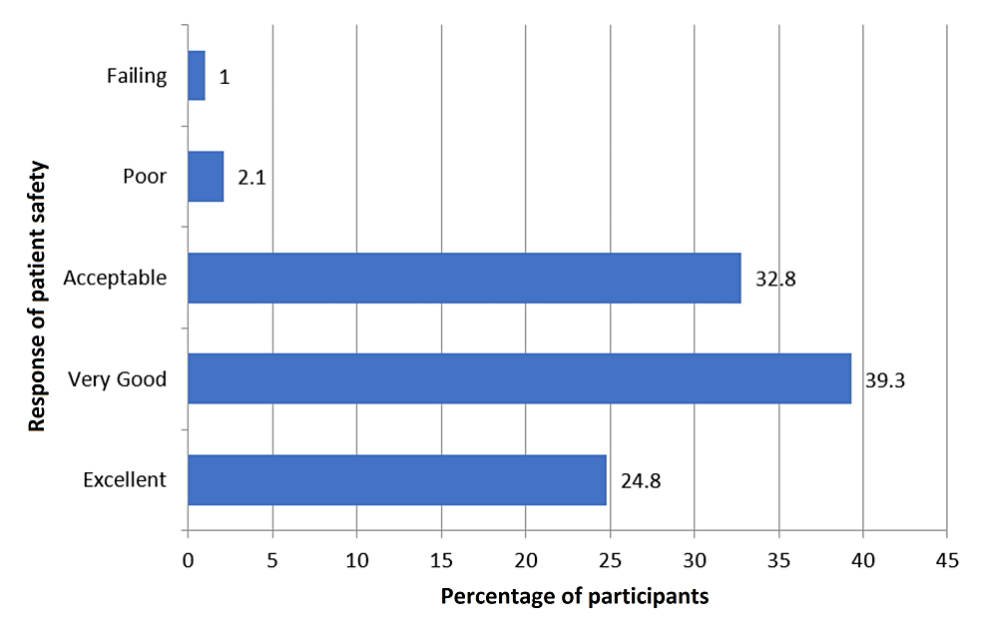
Percent positive scores about overall grades from all respondents on patient safety in public tertiary care hospitals.

**Figure 2:**
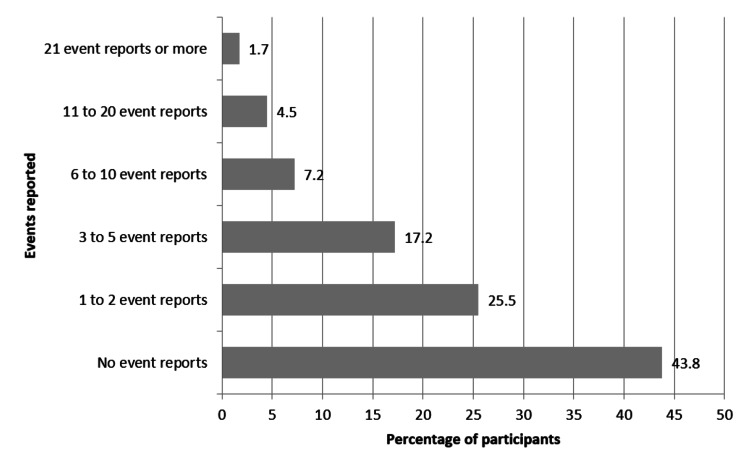
Percentage of number of events reported in past 12 months in public tertiary care hospitals.

Considering the overall grade on patient safety, 39.3% of respondents graded it as very good, followed by 32.8% as acceptable, 24.8% as excellent, 2.1% as poor, and 1% as failing (Figure 1). Most of the respondents (43.8%) indicated that no adverse event was reported while 25.5% of respondents reported one to two adverse events, 17.2% of respondents reported three to five adverse events, and 7.2% of respondents reported 6-10 adverse events. Further, 4.5% of respondents reported 11-20 adverse events and 1.7% of respondents reported 21 or more adverse events (Figure 2).

**Figure 3:**
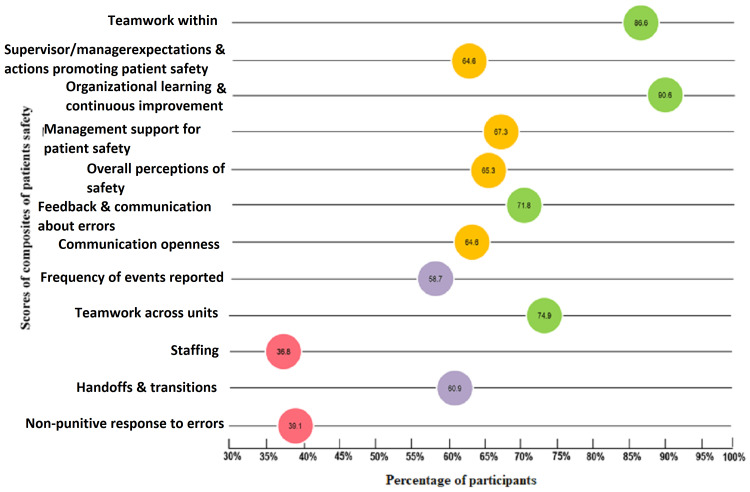
Overall percent positive scores of all 12 dimensions or composites of patient safety culture in public tertiary care hospitals.

With an overall 65.1% of PSC, the highest percent positive response rate was observed for organizational learning and continuous improvement (90.6%) followed by teamwork within units (86.6%), teamwork across units (74.9%), feedback, and communication about the error (71.8%), management support for patient safety (67.3%), overall perceptions of patient safety (65.3%), supervisor/manager expectations and actions promoting patient safety (64.6%), communication openness (64.6%), handoffs and transitions (60.9%), frequency of events reported (58.7%), and non-punitive response to errors (39.1%) and staffing (36.8%) (Figure 3).

## Discussion

The assessment of the existing patient safety practices and culture of the health system is the first stage of developing a positive patient safety culture (PSC). Perception of patient safety by healthcare staff can be assessed by the attitudes and beliefs of the clinical staff to raise their awareness and establish such practices that will further strengthen PSC. The present study is a primary investigation in Pakistan to highlight the significant glitches faced by healthcare practitioners in securing patient safety, improving patient care, and preventing adverse events during the delivery of healthcare services. In the present study, PSC was examined using its contributing factors, including twelve dimensions and personal variables. By reviewing the results, all dimensions of the overall perception of PSC had an average score of 65.3%. This reflects a need for improvement in safety standards and the implementation of corrective measures to enhance awareness of patient safety among healthcare professionals. The present study results agree with the findings of a 60% patient safety culture score in a public tertiary care hospital in Saudi Arabia [18]. The dimensions of organizational learning continuous improvement (90.6%) and teamwork within units (86.6%) were highest. It showed a strongly developed status while other dimensions including feedback and communication about the error (71.8%), teamwork across units (74.9%), management support for patient safety (67.3%), supervisor/manager expectations and actions promoting patient safety (64.6%), communication openness (64.5%), overall perceptions of patient safety (65.3%), frequency of events reported (58.7%), and handoffs and transitions (60.9%) showed moderate status. The dimensions of staffing (35.8%) and non-punitive response to errors (39.1%) had the lowest score.

The feedback and communication about errors had a 71.8% positive score which could be due to strict hierarchy maintenance, staff not wanting to complicate relationships among themselves, or non-responsiveness of higher authorities to report. Positive feedback and communication from working staff and administration about the error is vital to improving wrong practices, leading to an improvement in the PSC of tertiary healthcare centers [19,20]. The present study results showed that doctors and nurses perceived PSC more in the aspect of teamwork across units, which signified the impact of teamwork on the delivery of quality care and patient safety. This dimension of PSC has prime importance in delivering effective and safe care because treatment is usually done by a multidisciplinary team from different units within a hospital and may be helpful in eliminating threats to the safety of patients [21]. The present study's findings agree with the results observed in previous studies [19,21,22].

While comparing teamwork within units had a high positive score (86.6%). Similarly, a previous study found a higher positive score for teamwork within the unit (74.4%) than for teamwork across units (42.35%) [20]. The possible reason is that every unit has different goals to be achieved. Organizational and interpersonal barriers may hinder teamwork in the delivery of patient care and the hierarchical decision-making process [19]. A previous study has suggested that teamwork across units is more important than teamwork within units. Because teamwork and mutual help among team members to perform a task across different units using different instruments within a hospital may be an accurate representation of patient safety [23]. In the present study, the management support and supervisor or manager expectations and promotion of patient safety had moderate positive scores (67.3% and 64.6%, respectively). Similar findings were also observed during the assessment of PSC in public tertiary care hospitals in the Philippines and Saudi Arabia [21,24]. Clinical staff who worked closely together with supervisors or managers and supported by management in their work duties resulted in mutual respect and increased teamwork within and across units, leading to PSC enhancement [25]. A previous study in Canada revealed that management support and help from supervisors or managers might significantly enhance PSC by improving strategies and commitments in any healthcare organization [26].

The present study revealed that communication openness had a 64.5% positive score, which needs improvement for a strong PSC in public tertiary care hospitals across Lahore. The findings of the present study agree with the observation of the communication openness score (60.5%) reported in the United States [20]. On the contrary, communication openness showed a high positive score in Iranian and Dutch hospitals [27,28]. In contrast, communication openness was highlighted as an area of concern in Turkish and Kuwaiti hospitals with low positive scores [29,30]. A lack of communication among staff affects both safety cultures. It acts as a contributing factor to the incidence of adverse events and any human error, which needs to be addressed in strengthening PSC in any healthcare setting [28]. The present study revealed that organizational learning and continuous improvement had a positive score (90.6%). The present study's findings agree with the observation made by previous studies conducted in public tertiary care hospitals [24]. While comparing the outcomes of the present study, this variable is slightly improved from the previous survey conducted in Pakistan. The possible reason might be learning from assessment and preventing the same and other adverse events in a healthcare setting. In the present study, staffing had a low positive score of 35.8%, and most of the respondents indicated that they did not have enough staff to handle the workload. Such an inadequate response indicated a severe shortage of staff in public tertiary care hospitals. A similar outcome was also observed in a previous study conducted in the United States [20]. These outcomes showed that such a situation might have severe negative consequences for PSC and the delivery of quality care to patients. Because of less staff, the possibility of occurrence of falls and medication errors was increased due to increasing workload and unstable working environment [28].

Among all dimensions, the present study showed that non-punitive response to error had a very low positive score (39.1%). A previous study conducted in Pakistan also claimed that staff was scared to report mistakes and caused underreporting. The findings of the present study agree with the previous studies conducted in the United States and Sri Lanka with 21.09% and 39.4% positive responses, respectively [20]. The reason for the low positive score about the non-punitive response to the error indicates a strong blame culture that has prominent existence in the healthcare systems of Pakistan [14]. The present study results highlighted that medical staff perceive it to be punitive and are not supported or feel comfortable in reporting errors. This is not unusual because previous studies also observed that staff are afraid to report errors and feel threatened [18,21,30].

While interpreting the data, various limitations must be addressed. To begin, the sample size of 290 nurses and physicians from a single tertiary care hospital may not be indicative of the wider healthcare system in Lahore or of Pakistan as a whole. Second, the study lacks a comparison or control group, making it difficult to compare patient safety culture across different types of healthcare settings or evaluate the impact of specific treatments on enhancing patient safety culture. However, this methodology may fail to capture fluctuations and changes in patient safety culture over time, making it difficult to establish causal links or make clear conclusions regarding the impact of treatments.

## Conclusions

The present public hospital survey results revealed that medical staff working in healthcare settings across Lahore have a less positive perception of patient safety culture. Specific dimensions of PSC are those related to developing an effective error reporting system and non-punitive culture by allocating more staff that need significant improvement. The outcomes of this study could be used for designing and establishing interventions to improve patient safety practices across Pakistan. The use of cutting-edge technology to expedite mistake reporting procedures, the planning and implementation of extensive training initiatives to strengthen employee awareness, and the optimization of workforce numbers in areas in need of significant improvement should all be included in future directions to develop a uniform, national approach to patient safety, cooperation between academic institutions, regulatory agencies, and healthcare facilities should be encouraged. Furthermore, ongoing assessment, monitoring, and patient viewpoints being included in safety measures will be critical to interventions' long-term effectiveness. We can usher in a new age of patient safety by addressing these future directions, guaranteeing that healthcare delivery in Pakistan is characterized by a culture of excellence and continuous development in addition to being effective.

## References

[1] Hegarty J, Flaherty SJ, Saab MM (2021). An international perspective on definitions and terminology used to describe serious reportable patient safety incidents: a systematic review. J Patient Saf.

[2] Umachitra MN (2021). A study of unethical medical malpractices - a legal perspective. Ann Romanian Soc Cell Biol.

[3] Cole DA, Bersick E, Skarbek A, Cummins K, Dugan K, Grantoza R (2019). The courage to speak out: a study describing nurses' attitudes to report unsafe practices in patient care. J Nurs Manag.

[4] Li H, Dong S, Liao Z, Yao Y, Yuan S, Cui Y, Li G (2020). Retrospective analysis of medical malpractice claims in tertiary hospitals of China: the view from patient safety. BMJ Open.

[5] Reis CT, Paiva SG, Sousa P (2018). The patient safety culture: a systematic review by characteristics of Hospital Survey on Patient Safety Culture dimensions. Int J Qual Health Care.

[6] Alshehry AS (2019). Culture of quality in infection prevention of a hospital as perceived by health care workers. J Nurs Manag.

[7] Trinchero E, Farr-Wharton B, Brunetto Y (2019). Workplace relationships, psychological capital, accreditation and safety culture: a new framework of analysis within healthcare organizations. Public Organ Rev.

[8] Meddings J, Reichert H, Greene MT (2017). Evaluation of the association between Hospital Survey on Patient Safety Culture (HSOPS) measures and catheter-associated infections: results of two national collaboratives. BMJ Qual Saf.

[9] Andrade LE, Melo LO, Silva IG (2017). Adaptation and validation of the Hospital Survey on Patient Safety Culture in an electronic Brazilian version. Epidemiol Serv Saude.

[10] Waterson P, Carman EM, Manser T, Hammer A (2019). Hospital Survey on Patient Safety Culture (HSPSC): a systematic review of the psychometric properties of 62 international studies. BMJ Open.

[11] Okuyama JH, Galvao TF, Silva MT (2018). Healthcare professional's perception of patient safety measured by the hospital survey on patient safety culture: a systematic review and meta-analysis. ScientificWorldJournal.

[12] Sturm H, Rieger MA, Martus P, Ueding E, Wagner A, Holderried M, Maschmann J (2019). Do perceived working conditions and patient safety culture correlate with objective workload and patient outcomes: a cross-sectional explorative study from a German university hospital. PLoS One.

[13] Danielsson M, Nilsen P, Rutberg H, Årestedt K (2019). A national study of patient safety culture in hospitals in Sweden. J Patient Saf.

[14] Jafree SR, Zakar R, Fischer F, Zakar MZ (2015). Ethical violations in the clinical setting: the hidden curriculum learning experience of Pakistani nurses. BMC Med Ethics.

[15] Dayan F, Gulaly Gulaly, Sheraz MM, Zia-ul-Haq M (2020). Holding healthcare accountable: a solution to mitigate medical malpractice in pakistan. Int J Hum Health Sci.

[16] Shields MC, Stewart MT, Delaney KR (2018). Patient safety in inpatient psychiatry: a remaining frontier for health policy. Health Aff (Millwood).

[17] Sorra JS, Battles J (2014). Lessons from the AHRQ hospital survey on patient safety culture. Patient Safety Culture. First Edition.

[18] Alahmadi HA (2010). Assessment of patient safety culture in Saudi Arabian hospitals. Qual Saf Health Care.

[19] Coburn AF, Gage-Croll Z (2011). Improving hospital patient safety through teamwork: the use of TeamSTEPPS in critical access hospitals (policy brief #21). Policy Brief#21.

[20] Armellino D, Quinn Griffin MT, Fitzpatrick JJ (2010). Structural empowerment and patient safety culture among registered nurses working in adult critical care units. J Nurs Manag.

[21] El-Jardali F, Jaafar M, Dimassi H, Jamal D, Hamdan R (2010). The current state of patient safety culture in Lebanese hospitals: a study at baseline. Int J Qual Health Care.

[22] Chen IC, Li HH (2010). Measuring patient safety culture in Taiwan using the Hospital Survey on Patient Safety Culture (HSOPSC). BMC Health Serv Res.

[23] Boughaba A, Aberkane S, Fourar YO, Djebabra M (2019). Study of safety culture in healthcare institutions: case of an Algerian hospital. Int J Health Care Qual Assur.

[24] Ramos RR, Calidgid CC (2018). Patient safety culture among nurses at a tertiary government hospital in the Philippines. Appl Nurs Res.

[25] Hamdan M, Saleem AA (2013). Assessment of patient safety culture in Palestinian public hospitals. Int J Qual Health Care.

[26] Zaheer S, Ginsburg L, Chuang YT, Grace SL (2015). Patient safety climate (PSC) perceptions of frontline staff in acute care hospitals: examining the role of ease of reporting, unit norms of openness, and participative leadership. Health Care Manage Rev.

[27] Tabrizchi N, Sedaghat M (2012). The first study of patient safety culture in Iranian primary health centers. Acta Med Iran.

[28] Zwart DL, Langelaan M, van de Vooren RC, Kuyvenhoven MM, Kalkman CJ, Verheij TJ, Wagner C (2011). Patient safety culture measurement in general practice. Clinimetric properties of 'SCOPE'. BMC Fam Pract.

[29] Ghobashi MM, El-Ragehy HA, Ibrahim HM, Al-Doseri FA (2014). Assessment of patient safety culture in primary health care settings in Kuwait. Epidemiol Biostat Public Health.

[30] Bodur S, Filiz E (2009). A survey on patient safety culture in primary healthcare services in Turkey. Int J Qual Health Care.

